# Common Clinical and Molecular Pathways between Migraine and Sarcoidosis

**DOI:** 10.3390/ijms24098304

**Published:** 2023-05-05

**Authors:** Claudio Tana, David Garcia Azorin, Francesco Cinetto, Cesare Mantini, Marco Tana, Massimo Caulo, Fabrizio Ricci, Paolo Martelletti, Francesco Cipollone, Maria Adele Giamberardino

**Affiliations:** 1 Center of Excellence on Headache, Geriatrics and COVID-19 Clinic, SS Annunziata Hospital of Chieti, 66100 Chieti, Italy; 2 Headache Unit, Department of Neurology, Hospital Clínico Universitario de Valladolid, 47003 Valladolid, Spain; 3 Rare Diseases Referral Center, Internal Medicine 1, Ca’ Foncello Hospital—AULSS2 Marca Trevigiana and Department of Medicine—DIMED, University of Padova, 35122 Padova, Italy; 4 Department of Neuroscience, Imaging and Clinical Sciences, “G. D’Annunzio” University of Chieti-Pescara, 66100 Chieti, Italy; 5 2nd Internal Medicine Unit, SS. Medical Department, SS. Annunziata Hospital of Chieti, 66100 Chieti, Italy; 6 University Cardiology Division, Heart Department, SS. Annunziata Hospital of Chieti, 66100 Chieti, Italy; 7 Department of Clinical and Molecular Medicine, Sapienza University of Rome, 00185 Rome, Italy; 8 Medical Clinic, Department of Medicine and Science of Aging, SS. Annunziata Hospital of Chieti, “G. D’Annunzio” University of Chieti-Pescara, 66100 Chieti, Italy

**Keywords:** headache, migraine, sarcoidosis, molecular, biology, mechanisms, diagnosis, pain, morbidity, biopsy

## Abstract

Migraine and sarcoidosis are two distinct medical conditions that may have some common biological and clinical pathways. Sarcoidosis is a chronic granulomatous disease characterized by the formation of granulomas in various organs, including the lungs, skin, cardiovascular system, lymph nodes, and brain. Migraine is a common comorbidity in sarcoidosis patients and a common neurological disorder characterized by recurrent headaches that can be accompanied by other symptoms, such as nausea, vomiting, and sensitivity to light and sound. There have been several reports of individuals with neurosarcoidosis experiencing migraines, though the exact relationship between the two disorders is not well understood. Both conditions have been associated with inflammation and the activation of the immune system. In sarcoidosis, the formation of granulomas is thought to be an immune response to the presence of an unknown antigen. Similarly, the pain and other symptoms associated with migraines are thought to be caused by inflammation in the brain and the surrounding blood vessels. There is also evidence to suggest an interplay of environmental and genetic factors playing a role in both conditions, but evidence is inconsistent with the hypothesis of shared genetic susceptibility. This review aims to illustrate common clinical and biological pathways between migraine and sarcoidosis, including inflammation and dysregulation of the immune system, with a focus on the cumulative burden of concurrent disorders and therapeutic implications.

## 1. Introduction

Migraine is the third most prevalent disorder affecting the global population [1], and has a significant burden in terms of loss of work, impaired quality of life, and functioning by negatively impacting one’s personal, professional, and social life [2]. It represents the first cause of years lived with disability between 15–49 year olds, and its prevalence is highest in female subjects. It has been hypothesized that this group is affected mainly due to the hormonal mechanisms that occur during the childbearing age [3]. Sarcoidosis is a relatively uncommon condition characterized by persistent growth of granulomatous tissue inside organ and tissues, but most often prevails in sites such as lungs and intra thoracic lymph nodes. However, every site can be affected (e.g., skin, gastrointestinal system, liver, spleen, kidneys, and genitourinary tract, which are affected by the disease to a different degree) [4,5], and also the neurological system (both central and peripheral) is not spared from the disease. Neurosarcoidosis (NS) is a distinctive condition of extrapulmonary sarcoidosis in which the nervous system is mainly affected from granulomatous tissue, and headache is one of the main symptoms of overt disease. As it has been observed for migraine, women suffering from NS seem to be the group that is most often affected [6,7].

Some experimental data have also shown how sarcoidosis and primary headaches, in particular migraine, can correlate sometimes, both in molecular and clinical pathways [8]. 

The recent research focus on shared molecular mechanisms and the hypotheses of association between migraine and sarcoidosis are not interesting exclusively from a narrative point of view but also because they could be a good point of strategy to find common approaches in terms of the quantification of disease severity and treatment. In this review, a narrative search of public databases (PubMed, Scopus) of key terms, such as migraine AND sarcoidosis OR sarcoid lesions OR granulomatous tissue, or headache AND sarcoidosis OR sarcoid lesions OR granulomatous tissue, has been performed in order to find the articles with evidence on the correlation between the two disorders. The search was limited to studies written in the English language; both review articles and original studies were included.

## 2. Epidemiological Impact of Migraine and Sarcoidosis

### 2.1. Burden of Migraine in Terms of Work Loss and Impaired Quality of Life and Functioning

Migraine is one of the most frequent neurological conditions affecting people worldwide, and it is estimated that it affects 12% of the overall population (over 1 billion people worldwide), most frequently women [9,10]. The incidence of episodic pain is estimated to be higher, while a chronic pattern affects 1–2% of the population worldwide, with a conversion rate between episodic to chronic form of 2.5%, and several risk factor, including metabolic, have been considered as main triggers for chronicization [11]. The highest prevalence of migraine is observed in women; in 2019, it was estimated that migraine prevalence was around 17,902.5 per 100,000 people (95% UI: 15,588.3, 20,531.7) versus 10,337.6 (95% UI: 8948.0, 12,013.0) in men [12,13].

Interestingly, the disease prevails in women across all age groups, suggesting a strict correlation with some inhered conditions, such as hormonal imbalance (estrogen and cortisol), which has been hypothesized to be one of the most important mechanisms in this group of patients [14].

Migraine has a significant burden in terms of impaired quality of life and productivity loss. Social isolation and the feeling of loneliness are higher in patients with chronic migraine as opposed to patients with episodic migraine, isolation being the first action taken to reduce the pain. However, the tendency to isolate when migraine becomes chronic or resistant to analgesics or prophylaxis drugs can later be a warning sign for mood disorders [15]. Economic loss due to reduction in work productivity and employer costs is another great problem of patients with migraine that is refractory to treatment. Absenteeism for migraine was associated with an economic loss estimated recently to be around $238.3USD/year/person for days off and 90.2USD/year/person for half-days off using the migraine disability assessment score (MIDAS) in the Japanese population [16].

Approximately 60,000 to 686,000 annual workdays seem to be affected by lost productive time and absenteeism due to migraine, with higher indirect as compared to direct costs for the employers in United States [17].

Like other chronic conditions, the chronic self-perception of illness and pain and the reduction in work productivity and social isolation are associated with a significant rate of mood disorders and physiological disturbances, which in turn could worsen the headache symptoms, both in frequency and severity in a vicious circle [18].

### 2.2. Burden of Sarcoidosis and Headache-Related Pain

Unlike migraine, sarcoidosis is an uncommon disease that affects both sexes and all ethnicities, but reaches the highest incidence in white individuals, particularly those from northern countries and African Americans. Young patients have been identified as the category that is most often affected, but new data from a case control etiologic study of sarcoidosis (ACCESS) report a high incidence also after 50 age years, showing a biphasic curve of the disease [19,20,21,22]

The disease is more frequent in African Americans than white individuals, and this group of patients seems to have the most severe form of disease, in particular from cardiovascular involvement, which accounts for the highest risk of mortality. An early onset and family history are typical in African Americans and suggest the presence of inherited mechanisms in this group [21,23,24,25].

Like migraine, the burden of sarcoidosis can affect multiple categories, such as somatic, psychosocial, and economic. The somatic burden derived from organ symptoms, such as persistent headache, e.g., deriving from brain lesions, granulomatous infiltration, aseptic meningitis or symptoms related to small fiber neuropathy, characterized by a burning and shooting pain, could be really disabling and limit an individual’s daily routine [26,27]. The somatic burden also includes conditions such as physical impairment and loss of function due to the impairment of other organs, and could be researched accurately with surrogate markers of reduced capacity, with tests such as maximal oxygen consumption and 6 min walk distance for pulmonary disease [28,29]. These markers correlate well with reduced quality of life from physical dysfunction [21]. Additionally, cardiac involvement (e.g., heart failure and arrhythmias), eye disease with reduced long-term visual acuity, or hepatomegaly and gastrointestinal disease could significantly impact daily routine and functioning. Constitutional symptoms, such as fatigue, are also highly disabling in non-treated or non-responding patients, particularly if they are associated with persistent and unresponsive headache [30,31].

Psychosocial elements, such as depression-related pain, cognitive dysfunction, and fatigue, should be carefully investigated. The fact that the chronicity of pain, such as the persistence of unresponsive headache, could aggravate previous psychiatric disturbances in a vicious cycle has been documented [32].

The development of therapeutic interventions for psychosocial comorbidities, such as specific exercises, could give ameliorate the impact of pain and mood disorders [21].

The costs of sarcoidosis and pain related to headache are different depending on the involvement of different parties, such as patients, employers, caregivers, and governments. High direct costs are related to an increase in hospitalized patients (more than 80,000 in 2011; twice those in 1998), immunosuppressant drug and cortisone use, and imaging techniques, such as contrast-enhanced computed tomography (CECT) and positron emission tomography (PET). A reduction in work capacity, productivity, and income are the most important indirect costs that involve patients [21].

## 3. Headache in Migraine versus Sarcoidosis

### 3.1. Characteristics of Migraine

The International Classification of Headache (ICHD-3) defines migraine as a primary headache characterized by the presence of at least two characteristics, such as unilateral location, pulsating quality, moderate-to-severe pain intensity, and aggravation from routine physical activity, and at least one symptom between nausea and/or vomiting and photo/phonophobia during a migraine attack. Sometimes migraine occurs after temporary visual or other clinical manifestations [33]. Migraine is a chronic and benign disorder, and diagnosis is usually made clinically in accordance with the ICHD-3 criteria. MRI is recommended only for patients who present with changes in the headache phenotype, frequency or severity, or any other atypical symptom (red flags of secondary headache). Positron emission tomography and computed tomography (PET-CT) has been used for research purposes only but has no indication for the daily routine diagnosis of migraine [33].

### 3.2. Migraine in Patients with Sarcoidosis

To date, the best evidence about the association between sarcoidosis and migraine was obtained from a cohort study that included 126 subjects with sarcoidosis and 64 controls between January 2010 and May 2015. The frequency of migraine, diagnosed by the ID migraine screening [34], was 22/78 (28%) in patients with sarcoidosis without NS; 6/18 (33%) in patients with sarcoidosis with NS, and 5/39 (13%) in unaffected controls [8]. In a univariate regression, the only variable that was associated with the diagnosis of migraine was the female sex (OR: 2.7; 95% CI: 1.02–6.84). A few case reports have described patients with sarcoidosis and a migraine-like headache [35], while in other cases, the description of the headache does not allow its classification [36,37,38,39]. Sarcoidosis could be another comorbidity of migraine, since its frequency seems to be twice as high as what is expected to occur by chance [11]. As it has been discussed for migraine, patients with a migraine-like headache that report clinical red flags should be examined with MRI, since headache may be the symptom of another neurological condition, such as pachymeningitis or intracranial hypertension [40,41].

### 3.3. Headache as Clinical Manifestation of NS

NS is the first and main clinical manifestation in 5–10% of patients with sarcoidosis, but it is found in up to 25% of autopsies, suggesting its great difficulty in being identified in a routine clinical approach. Unlike migraine, which manifests only with headache, NS affects both the central and peripheral nervous systems, and myopathy is classified among the neuromuscular manifestations of NS. The involvement can be isolated or associated (more often) with other manifestations of the disease, such as pulmonary disease [26]. The mean onset age is later than in other forms of sarcoidosis (33–41 years), but neurological symptoms are clinically overt since the first two years of diagnosis [42].

Some series report the highest prevalence of NS in black people and women as for other forms of sarcoidosis [43]. However, not all studies agree, indeed Caucasians were most often affected in a French study population (91% of patients) [44], and the male sex was reported to be significantly affected in a case study from the United Kingdom [45].

Headache is the second most frequent manifestation of NS. In a meta-analysis that analyzed 29 articles and included 1088 patients diagnosed between 1965 and 2015, headache was the second most common manifestation, affecting 31% (95% CI: 28–35%) patients [46]. Headache is usually characterized by a tension-like form [47] and could be classified among the secondary headaches; the 7.3.1 group of the International Classification of Headache (ICHD-3) states that “headache attributed to non-vascular intracranial disorders/noninfectious inflammatory intracranial disease/headache attributed to NS” and the diagnosis is based on the temporal relationship between the diagnosis of NS and headache onset [33].

However, the clinical presentation can be different; some authors have indeed found that headache had the clinical features of Tolosa–Hunt syndrome in 50% of NS patients in their study, citing as evidence a lesion in the cavernous sinus [47]. The lesion could be associated with cranial neuropathy, as the most common clinical presentation of NS, present in approximately 55% of patients and manifests usually with the characteristics of painful paralysis of the third cranial nerve, although any nerve of the cavernous sinus can be affected [46]. Headache, therefore, can manifest differently according to the neuropathologic involvement, and a detailed imaging study is recommended along with a cerebrospinal fluid (CSF) examination to achieve the correct diagnosis and treatment approach [47]. Biopsy is less frequently needed; it is most often required in isolated forms where a radiological picture and a liquor analysis are not conclusive, or in those cases that mimic tumors and a correct diagnosis is needed before starting an appropriate therapy (corticosteroid treatment for sarcoid lesions, surgery or chemotherapy for tumors) [48].

Headache can be chronic in some patients, and refractory forms seem to respond well to cortisone treatment [49].

Other non-specific symptoms of NS are fatigue, which seems to be associated with cognitive dysfunction in NS patients, low-grade fever, mood disorders, such as depression, and nausea and/or vomiting, which can complete the clinical picture. A detailed presentation of the clinical features of NS is presented in Table 1 [26].

## 4. Imaging Diagnosis of NS

### 4.1. Magnetic Resonance Imaging

Magnetic resonance imaging (MRI) is an important diagnostic tool for the first evaluation of individuals with NS, and for monitoring them over the follow-up [50,51,52]. Follow-up duration can vary on the basis of the onset of new symptoms and disease severity, and a close monitoring may be required in patients with a more severe disease. However, an easier follow-up approach is reached with whole-body 18F-fluorodeoxyglucose (18F-FDG) PET-CT, and to monitor responses to therapy (see below).

**Figure 1:**
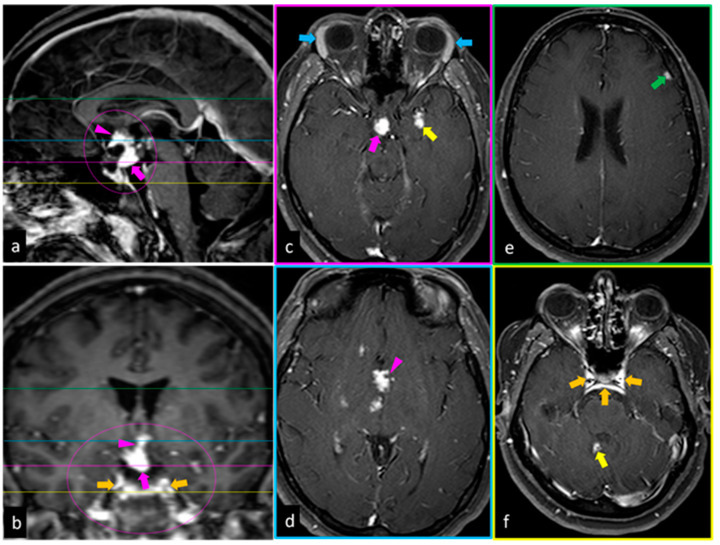
Ref. [53] A case of NS. Sagittal (**a**), coronal (**b**), and axial (**c**,**d**) post-contrast T1-weighted MR images show an extensive enhancement of the pituitary gland (pink arrows) and stalk (pink arrowheads), which is markedly enlarged. Both diffuse and nodular patterns are evident. A small extra-axial enhancing mass of dura is found (green arrow in (**e**)). Coronal (**b**) and axial (**f**) post-contrast T1-weighted MR images show the widespread thickening and enhancement of the leptomeninges (orange arrows) along the convexities of the brain, near the basal cisterns. Contrast-enhanced T1-weighted axial MR image shows enhancing parenchymal mass lesions in the left temporal lobe (yellow arrow in (**c**)) and in the cerebellum (yellow arrow in (**f**)), and bilateral enlarged lacrimal glands (light blue arrows in (**c**)).

The most common areas of involvement that are discovered with MRI are:

- Intraparenchymal portion of the brain (Figure 1c,f) and spine (most common);
- Leptomeningeal area (Figure 1b,f);
- Pachymeningeal area (Figure 1e);
- Pituitary gland and hypotalamus (Figure 1a–d);
- Cranial nerve roots [53].

It is important, therefore, to detect any signs of nervous system involvement early. The appearance of NS lesions is often nonspecific and differential diagnosis with other mimicking lesions is mandatory (e.g., malignant lesions, other granulomatous disorders, infectious diseases, abscesses, foreign bodies etc.).

NS frequently occurs with enhancing parenchymal mass lesions (35% of cases developing as multiple infratentorial and/or supratentorial masses; 15% as solitary masses) (Figure 1c,f), frequently associated with nearby leptomeningeal involvement (Figure 1b,f) [54]. The MRI differential diagnosis includes metastatic disease, demyelinating disease, and gliomas.

Forty percent of patients with NS had leptomeningeal involvement, and typically, the basilar meninges are affected [55]. This finding is characterized by enhanced and thickened leptomeninges on post-contrast T1-weighted MR images (Figure 1b,f); the thickening may be nodular or diffuse. The involvement of the perivascular spaces, cortical sulci, and the cisterns at the base of the brain can help to differentiate leptomeningeal disease from the dural. The MRI differential diagnosis includes lymphomatous meningitis, carcinomatous meningitis, and infectious meningitis.

In 34% of patients with NS, the dura is involved [56]. This finding is seen as focal hyperintense dural masses (Figure 1b,f) or diffuse hyperintense dural thickening on post-contrast T1-weighted MR images. The MRI differential diagnosis includes meningioma, metastasis, and infectious meningitis.

In a small percentage (18%) of NS patients, the pituitary gland or hypothalamus are affected, alone or in association with basilar leptomeningeal involvement, which appear thickened and enhanced on post-contrast T1-weighted MR images [56]. This finding is seen mostly as thickening and enhancement on contrast-enhanced T1-weighted images. The MRI differential diagnosis includes some disorders, such as lymphoma, tuberculosis, Langerhans cell histiocytosis, and metastases.

### 4.2. PET-CT

18F-FDG PET-CT is very useful in identifying a correct site for biopsy in the central nervous system, and therefore, confirming the diagnosis of NS [57,58]. The biopsy is highly recommended when the involvement of the nervous system is isolated. In patients with negative or inconclusive chest CT and when central nervous system biopsy is not possible or complex, 18F-FDG PET-CT is useful to give a “probable” NS diagnosis.

Another field of application of 18F-FDG PET-CT is the evaluation of response to the treatment, and to monitor patients during the follow-up time. An effective therapeutic approach is documented as the improvement of imaging findings at the follow-up, and the shorter re-evaluation time could be needed for aggressive forms and non-responsive disease [59,60]

## 5. Shared Molecular Mechanisms between Migraine and Sarcoidosis

The immunopathogenesis of sarcoidosis has been extensively studied but it is still far from being completely understood [61]. As far as CNS involvement is concerned, in particular, data on the peculiar characteristics of granulomatous inflammation in this specific microenvironment are still limited due to the rarity of NS, whose diagnosis is often made without direct histologic confirmation [62]. In lack of large studies on histologic samples and since specific experimental models are not available in the literature, data from plasma and CSF-based studies in patients with NS provide most of the available information on the systemic and local signatures of this peculiar sarcoidosis manifestation [63]. Differently from NS, migraine is not a rare disease. However, it is characterized by ictal episodes and interictal phases, and diagnostic approaches are generally less invasive compared to NS, thus making it harder to capture the signals of a specific immunologic microenvironment, ongoing at the CNS level. On the other hand, pre-clinical and clinical experimental models have significantly contributed, over the last few years, to providing interesting insights into the brain structures that mediate migraine attacks, also leading to the development of novel therapeutic approaches [64].

A recent study on 20 patients with NS and 11 healthy controls showed that 80% of the NS patients presented CSF pleocytosis, with T cells representing more than 88% of the white blood cells and a median CD4/CD8 ratio of 3.3. On the contrary, peripheral blood lymphocytes were significantly lower in NS patients compared to healthy control. Interestingly, the most frequent symptom in this NS group was headache (60%), followed by vertigo (55%) and tinnitus (50%) [65]. Peripheral T cell exhaustion and dysfunctional Treg cells have been implicated in the pathogenesis of sarcoidosis [61]. No pleocytosis has instead been reported in the CSF of migraine patients, ictally or interictally [66]. In the peripheral blood of a cohort of children and adolescents suffering from migraine without aura, migraine with aura, and hemiplegic migraine, CD8+ prevalence was lower, and the CD4+/CD8+ lymphocytes ratio was higher in the ictal phase irrespective of the subtype of migraine [67].

Interestingly, significant changes in the CD4+ effector memory T cells and terminally differentiated CD8+ T lymphocytes have been observed in migraine patients without aura, despite the interictal phase, with possible implications on disease severity [68]. A decreased level of Treg cells was also detected in migraine patients, particularly in the ictal phase and regardless of the migraine subtypes [69]. This finding was confirmed in subsequent studies [70]. Consistently, a pre-clinical study using a headache mouse model showed that triggering migraine-like headache by nitroglycerine (NTG, the most widely accepted nitric oxide donor) administration resulted in increasing CD3+ cell infiltration in the tri-geminal ganglion. Moreover, a repeated administration of low-dose interleukin-2 (IL-2) was able to induce an increase in Treg cells and prevent NTG-induced behavioral changes, while this was not possible in Treg-depleted mice [71]. It was subsequently demonstrated that low-dose IL-2 acts through IL-10 and TGF-β signaling [72]. All these considered, the role of T lymphocytes in the pathogenesis of migraine appears to be well supported. The specific role of Tregs in migraine is still under investigation, particularly regarding the possible use of the peripheral Treg cell population as a potential therapeutically relevant diagnostic biomarker for migraine [73].

Moving from lymphocytes to cytokines and other mediators, of the 16 investigated cytokines in the above-mentioned cohort of NS patients, 9 (IFN-γ, TNF-α, TNF-β, IL-2, IL-6, IL-10, IL-12B, IL-15, and IL-16) were found to have been significantly increased in the CSF of NS patients compared to controls, IFN-γ and IL-12B being those with the highest increase. Only IFN-γ and TNF-α concentrations were also significantly increased in plasma compared to healthy controls [73]. Another study reported IL-6 CSF levels to be correlated with disease activity and prognosis [63]. In a recent study enrolling patients with migraine with and without aura, the peripheral blood expressions of IFN-γ and TNF-α, as well as IL-4 and TGF-β were higher than in healthy controls [74].

A number of chemokines was also found to be higher in the CSF of NS patients, as well as VEGF-A between vascular angiogenesis markers and PIGF, SAA, VCAM-1, and ICAM-1 between the investigated injury biomarkers. Vascular cell adhesion molecule-1 VCAM-1 and intracellular adhesion molecule-1 (ICAM-1), in particular, were found elevated both in the CSF and plasma of NS patients, likely due to their role in leukocyte adhesion and transmigration, suggesting a role of endothelial activation and dysfunction in the pathogenesis of NS [73]. Interestingly, endothelial dysfunction with the increase in the serum levels of the intracellular adhesion molecules (ICAM) and vascular cell adhesion molecules (VCAM) has been also demonstrated in children and young adults with migraine [75]. sVCAM-1 levels have also been found significantly higher in subjects with more frequent migraine; in the same study, IL-6 was the only inflammatory mediator that was found to be higher in migraine patients than controls, when adjusting for age and sex, but without reaching statistical significance [65]. Notably, the decrease in VCAM-1 serum levels has been correlated to the therapeutic efficacy of alpha-lipoic acid (ALA) supplementation, used as an adjunct treatment in a clinical trial involving a population of female migraine patients without aura. [76].

Finally, in patients with acute migraine attacks, higher serum calcitonin gene-related peptide (CGRP) and pentraxine-3 (PTX-3) levels have been reported compared to controls [77,78]. CGRP has not been specifically investigated in sarcoidosis, but pentraxin-3 has been recently identified as a key regulator of the progression of granulomatous inflammation, despite no specific data being available for NS [79].

In conclusion, in lack of specific data regarding the immunopathogenesis of migraine in sarcoidosis and the possible mechanistic association between the two diseases, current evidence supports the involvement of lymphocytes dysregulation and Tregs dysfunction both in the pathogenesis of NS and migraine. Moreover, the available data from CSF and plasma studies suggest that the involvement of innate immunity and endothelial dysfunction may be another common feature. However, further studies are required to better highlight similarities and differences in terms of mechanisms and responses to possible therapeutic approaches.

## 6. Common Ways of Treatment and Strategies of Approach on Disease Severity

The treatment of secondary headache disorders is commonly based on the clinical phenotype of the headache [80]. The acute treatment of migraine is based on non-steroidal anti-inflammatory drugs and triptans [81]. Paracetamol is not recommended in the acute treatment of migraine since the probability of response is far lower than with other acute drugs [82]; however, it can be used in special populations and patients with comorbidities or contraindications, and its use can be attempted in patients with sarcoidosis as well. Patients with secondary headache disorders may respond to acute medications [83].

Patients with comorbid migraine should be treated as any other migraine patient. In the case of preventive treatments, some preventive drugs, such as amitriptyline, may be beneficial for migraine, tension-type headache, and other secondary headache disorders [84,85]. Other preventive drugs can be used, with special caution in the case of beta-blockers, in patients with severe pulmonary involvement [86].

Corticosteroids, metothrexate, azatioprine, miycophenolate mofetil, and anti-tumoral necrosis factor are the main treatments for NS [87,88]. In a study that included 56 patients who were evaluated between 2010 and 2018, 21% of them with headache, the proportion of patients who responded to treatment was higher in patients treated with infliximab (45%), followed by azathiprine (38%), prednisone (37%), and metotrexate (19%) [51]. In a study that compared the relapse rate of 40 patients treated with methotrexate and mycophenolate mofetil, a relapse rate of 47% and 79%, respectively, was reported; the median time of relapse in patients treated with mycophenolate was also shorter [89].

In a study that included 66 patients with central nervous system (CNS) sarcoidosis, clinical or radiological improvement was observed in 77.3% and 82.1% of patients, respectively [90]. In another study that included 28 patients with CNS sarcoidosis, including 10 patients with headache, showed that 71% of patients improved, allowing the tapering or discontinuation of corticosteroids in 68% of patients [91].

In another study that included 18 patients with NS, including 16/18 (88.8%) with prior use of other therapies, 16/18 (88.8%) patients improved after infliximab therapy [92].

Headache may also improve following disease-modifying therapies. In a study that reported on seven patients with NS, four of them with headache as a symptom, headache improved in all patients following cortoicosteroids, mycophenolate or infliximab [93].

Headache is one of the most frequent adverse effects of the treatments [50,94] used in sarcoidosis and NS patients, and it may be a symptom of systemic and intracranial infections, which are the most frequent complication of sarcoidosis therapies.

## 7. Conclusions

Migraine is a common comorbidity in patients with sarcoidosis and more often so in women with NS. Both conditions have been associated with the chronic inflammation and dysregulation of the immune system, and with significant cumulative somatic, psychosocial, and economic burden. The two disorders seem to share some pathological mechanisms, which in part are related to persistent inflammation and immune dysregulation, but they are largely unknown. So far, very few studies have investigated the molecular mechanisms connecting these diseases, and most research is based on retrospective design studies including a very limited sample size of patients. A clear delimitation between migraine and sarcoidosis is difficult, and further prospective research including larger sample sizes is needed to understand the complex relationship between them. A better awareness of the molecular mechanisms underlying the link between the two disorders could be useful in developing effective treatment options for the individuals affected.

## Figures and Tables

**Table 1:** Main clinical features of neurosarcoidosis (NS) [26].

Central Nervous System Involvement	Peripheral Nervous System Disease	Myopathy
Non-specific symptoms (headache, fatigue, cognitive dysfunction with decline, fever, nausea and vomiting, mood disorders). Cranial neuropathy (II, III, VI, VII nerve involvement). Seizures and focal neurological deficits (hemiparesis) from brain tumor-like masses. Endocrine dysfunction (diabetes insipidus, hyperprolactinemia, TSH or gonadotropin deficiency). Ischemic or hemorrhagic stroke with focal deficits. Involvement of spinal cord, most often thoracic, with paresthesia and lower extremities weakness.	Mono- or multifocal neuropathy with or without conduction blocks. Poly-radiculoneuropathy (Guillain–Barre’-like syndrome). Asymmetrical sensory motor polyneuropathy. Less frequently patterns: atypical chronic inflammatory demyelinating polyneuropathy, small-fiber neuropathy, or involvement of autonomic fibers with pain or restless leg syndrome.	Non-specific, pain, muscle weakness, and atrophy. Acute myositis with fever, fatigue, disabling pain, muscle swelling, and sometimes contractures. Chronic myositis presenting as multiple tumor-like nodules found on physical examination.

## Data Availability

Not applicable.
